# Acetaminophen-induced hypotension in the surgical ICU

**DOI:** 10.1186/cc13355

**Published:** 2014-03-17

**Authors:** Z Stoecker

**Affiliations:** 1 Hennepin County Medical Center, Minneapolis, MN, USA

## Introduction

Acetaminophen (APAP) is commonly administered in the surgical ICU (SICU) for its analgesic and antipyretic effects. Case reports have described the potential for APAP to cause allergic reactions with and without hypotension. Furthermore, there have been case reports of APAP causing isolated hypotension in the absence of other allergic responses [1]. This has been well described following intravenous administration [2]. However, other routes of administration causing hypotension and associated diagnoses remain to be elucidated. The present case series describes 11 patients with APAP-induced hypotension in the SICU at a Level I trauma center.

## Methods

Patients admitted to the SICU over a 7-month time frame who were reported by the nursing staff to have experienced hypotension following the oral or rectal administration of APAP were included. Their electronic medical records were retrospectively reviewed to describe the change in systolic blood pressure (SBP) and mean arterial pressure (MAP) within the first hour following all administrations of APAP. Additional data collected consisted of patient age, sex, admission diagnoses and formulation/route of APAP administration.

## Results

Of the 11 patients included, six had spontaneous intracranial hemorrhage (ICH), four had traumatic ICH and one was free of neurologic injury. Following administration of 393 doses of APAP, the average change in MAP for all patients was -7.52 mmHg and SBP was -12.04 mmHg. When evaluated on an individual basis, patients with ICH had an average change in MAP of -10.64 (-5.2 to -19.08) and SBP of -17.81 (-7.54 to -35.75). The patient without neurologic injury had an average change in MAP and SBP of -3.01 and -2.42. The average change in MAP and SBP following administration of oral APAP solution (*n *= 357) was -7.84 and -12.39 while the change following rectal administration (*n *= 3) was -4.22 and -8 and tablet administration (*n *= 33) was -4.4 and -8.61.

## Conclusion

It appears from this small case series that patients with ICH may experience more isolated hypotension following administration of ApaP when compared with others. These changes seemed to be greater than previously reported for intravenous administration. It may also be possible that APAP solution exudes hypotensive effects more commonly when compared with tablet or rectal formulations.
